# A remotely sensed flooding indicator associated with cattle and buffalo leptospirosis cases in Thailand 2011–2013

**DOI:** 10.1186/s12879-018-3537-3

**Published:** 2018-11-29

**Authors:** Sudarat Chadsuthi, Karine Chalvet-Monfray, Anuwat Wiratsudakul, Duangjai Suwancharoen, Julien Cappelle

**Affiliations:** 1 Department of Physics, Research Center for Academic Excellence in Applied Physics, Faculty of Science, Naresuan University, Phitsanulok, 65000 Thailand; 2 Université Clermont Auvergne, Université de Lyon, INRA, VetAgro Sup, UMR EPIA, 63122 Saint Genès Champanelle, France; 3 Department of Clinical Sciences and Public Health, and the Monitoring and Surveillance Center for Zoonotic Diseases in Wildlife and Exotic Animals, Faculty of Veterinary Science, Mahidol University, Nakhon Pathom, 73170 Thailand; 4 National Institute of Animal Health, Department of Livestock Development, Bangkok, 10900 Thailand; 5 ASTRE, CIRAD, INRA, Université de Montpellier, 34398 Montpellier, France; 6 CIRAD, UMR ASTRE, 34398 Montpellier, France

**Keywords:** Leptospirosis, Flooding, Buffalo, Cattle, Thailand, Satellite imagery

## Abstract

**Background:**

Leptospirosis is an important zoonotic disease worldwide, caused by spirochetes bacteria of the genus *Leptospira*. In Thailand, cattle and buffalo used in agriculture are in close contact with human beings. During flooding, bacteria can quickly spread throughout an environment, increasing the risk of leptospirosis infection. The aim of this study was to investigate the association of several environmental factors with cattle and buffalo leptospirosis cases in Thailand, with a focus on flooding.

**Method:**

A total of 3571 urine samples were collected from cattle and buffalo in 107 districts by field veterinarians from January 2011 to February 2013. All samples were examined for the presence of leptospirosis infection by loop-mediated isothermal amplification (LAMP). Environmental data, including rainfall, percentage of flooded area (estimated by remote sensing), average elevation, and human and livestock population density were used to build a generalized linear mixed model.

**Results:**

A total of 311 out of 3571 (8.43%) urine samples tested positive by the LAMP technique. Positive samples were recorded in 51 out of 107 districts (47.66%). Results showed a significant association between the percentage of the area flooded at district level and leptospirosis infection in cattle and buffalo (*p* = 0.023). Using this data, a map with a predicted risk of leptospirosis can be developed to help forecast leptospirosis cases in the field.

**Conclusions:**

Our model allows the identification of areas and periods when the risk of leptospirosis infection is higher in cattle and buffalo, mainly due to a seasonal flooding. The increased risk of leptospirosis infection can also be higher in humans too. These areas and periods should be targeted for leptospirosis surveillance and control in both humans and animals.

**Electronic supplementary material:**

The online version of this article (10.1186/s12879-018-3537-3) contains supplementary material, which is available to authorized users.

## Background

Leptospirosis is an important worldwide zoonotic disease, caused by spirochetes bacteria of the genus *Leptospira* [1, 2]. This bacteria is classified into pathogenic and nonpathogenic species, with more than 250 pathogenic serovars [1–3]. The disease is particularly important in tropical and subtropical countries. Human and animal infections can occur through direct exposure to infected animals or to indirect exposure to the soil or water contaminated with urine from an infected animal through skin abrasions or mucous membranes [1, 2].

In livestock, it is considered one of the most important diseases, particularly in cattle due to reproductive failures (such as abortion, embryonic death, stillbirths and weak off-spring), decreased milk production and growth rates [1, 4–6]. This results in significant economic losses [7] given the importance of these animals in tropical countries. In Thailand, about 4.4 million beef cattle, 0.51 million dairy cattle, and 0.89 million buffaloes were raised by 770,000, 160,000 and 200,000 households in 2012, respectively [8]. In rural areas, cattle and buffalo live in close contact with agricultural workers, and can be a major source of leptospirosis in humans, as highlighted by the predominance of the same serovars in both livestock and humans [4, 9]. Furthermore, a relatively high prevalence of leptospirosis have been detected in the urine of cattle and buffalo in Thailand [10]. An important route of transmission of *Leptospira* from livestock to humans could then be through contaminated urine [1, 2]. And as a consequence, flooding may be an important factor facilitating the transmission of *Leptospira* from livestock to humans and other animals by facilitating the spread of bacteria in wet soils and surface water, where the bacteria can survive for several weeks or months [11].

In humans, the number of reported leptospirosis cases in Thailand is highest after the peak in the rainy season [12]. Higher numbers of leptospirosis cases have been reported following rain or flooding in tropical and subtropical areas (e.g., Laos [13], Guyana [14], and Sri Lanka [15]). In Thailand, most reported cases occurred in northern and northeastern regions, where the main occupation is rice farming. Agricultural workers are the most exposed to biological contaminates in the environment. A previous study in Thailand found that human leptospirosis infections were observed near rivers, and mostly in rice fields likely to have flooding [16]. Furthermore, heavy rain and flooding have been identified as environmental drivers of leptospirosis infections in animals [17]. In the same way, leptospirosis infection risk is associated with flooding in Laos, particularly for human beings who have behaviors and activities involving contact with floodwater [13]. Overall, flooding appears as an important driver of leptospirosis infection in both humans and animals. By taking into account the seasonal variations of flooding using remotely sensed indicators, it may help in anticipating the risk of leptospirosis infection and identify periods and areas for increased surveillance and prevention [18].

The main objective of this study was to investigate the association of several environmental factors (especially remotely sensed indicators of flooding) with cattle and buffalo leptospirosis cases in Thailand. A model of leptospirosis infection risk at the district level was produced, taking into account seasonal flooding.

## Materials and methods

### Epidemiological data

A total of 3571 urine samples derived from 488 buffalo and 3083 cattle, were collected from January 2011 to February 2013 under a cross-sectional program, which has been described in detail in a recent article [4]. The sampling process was prepared by the provincial Department of Livestock Development livestock officers in 107 districts from 28 provinces, and the samples were randomly selected from each region of Thailand [4]. The sample size was calculated using the multi-stage clustered sampling technique. Three provinces in each of the 9 livestock administrative regions were chosen to represent the area. Subsequently, districts within the provinces were sampled. The target sample size in each region was calculated with the method proposed by Yamane [19]. In this study, we combined 9 regions of Thailand into 4 parts with different climate and seasonal flooding patterns, i.e. the Northern part, subdivided into the Upper Northern and Lower Northern, Central part, which consists of Central, Western and Eastern sub-regions, Northeast part, which consist of Upper Northeastern and Lower Northeastern regions, and the South, which consist of Upper Southern and Lower Southern regions. In their study, the number of samples in each district was not controlled. Sampling was not systematically repeated in all districts, but data was collected during the whole year in the different districts. All urine samples were examined for the presence/absence of leptospiral infection by loop-mediated isothermal amplification (LAMP) method [4, 10]. This technique showed high sensitivity and specificity at 96.8 and 97.0%, respectively [10].

### Environmental data

The environmental variables tested in our study include rainfall, flooded area, elevation, and human and livestock population densities. Flooding is an important driver of leptospirosis, but no data is readily available. The flooding variable was calculated based on the modified normalized difference water index (MNDWI). Other variables were collected from national or international databases. All variables were aggregated at the district level to match the spatial resolution of the epidemiological data.

The amount of rainfall was obtained from near Real-time TRMM (Tropical Rainfall Measuring Mission) multi-satellite precipitation analysis (TMPA-RT), which is produced at the National Aeronautics and Space Administration, Goddard Earth Sciences Data and Information Services Center (NASA GES DISC) [20]. The daily accumulated precipitation product is generated from the Near Real-Time Precipitation 3-hourly 1 day TMPA at a spatial resolution of 0.25 degree × 0.25 degree Version 7 (TRMM 3B42RT Daily) [21, 22]. In this study, given the homogeneity of rainfall at the district level, we only extracted the TRMM data at the centroid of each district.

To identify flooded areas, we used the data from the Moderate Resolution Imaging Spectroradiometer (MODIS) of the Terra satellite (Surface Reflectance 8-Day L3 Global 500 m SIN Grid V005 (MOD09A1)). In each image pixel, the data provides an estimation of the surface spectral reflectance measured at ground level in the absence of atmospheric scattering or absorption. The band 4 (green) and band 7 (infrared) were used to calculate the modified normalized difference water index (MNDWI) [18, 23], which allows an estimate of the water presence in each pixel. Within all districts, each pixel was classified as flooded if the MNDWI value was more than or equal to zero. This threshold of zero for MNDWI is in the range of optimal thresholds calibrated in previous studies [23–25]. Permanent water bodies such as rivers and lakes were masked out using QGIS version 2.8.3 [26]. Then, the number of flooded pixels were counted to calculate the percentage of flooded land in each district.

Elevation can be associated with slopes and increased movement of surface water [27], but slope data was not available at a national scale in Thailand. Elevation data was derived from the NASA Shuttle Radar Topographic Mission (SRTM) 90 m Digital Elevation Data, which provides elevation data for the entire world (http://srtm.csi.cgiar.org/index.asp). The average elevation at the district level was used in the model.

Human population data was obtained from the WorldPop database, which presents the number of people per hectare (http://www.worldpop.org.uk) (Additional file 2: Figure S5). Human population density was included in the model because it could be associated with different agricultural practices in areas with different levels of economic development. The animal population density of livestock species (buffalo, cattle, goat, pigs and sheep) were obtained from the Information and Communication Technology Center (ICT), Department of Livestock Development of Thailand at the district level (http://ict.dld.go.th) (Additional file 2: Figure S5). Goats, pigs and sheep were included because they may also contribute to the circulation of leptospirosis in cattle and buffaloes. Seroprevalences of other livestock were shown in Thailand from January to August 2001 in a previous study [28]. In this study, no urine samples were collected in urban districts because limited number of cattle and buffaloes are found in areas of high human population density. The districts with a human population density above 1400 people/km^2^, which corresponds to the urban centers of the main cities of Thailand, and no livestock were not included in the risk mapping given the limited number of animals in urban centers.

### Statistical analysis

To investigate the association between the risk factors listed in the previous paragraph (explanatory variables with a fixed effect) and leptospirosis infection (the response variable), we first study univariate linear regressions. Using a generalized linear mixed model (GLMM) with a logit link since the response variable had a binomial distribution. We used R software [29] with the package lme4 [30]. Since all individual urine samples were not independent because they were collected during common sampling occasions, we used the sampling occasion index as a random effect variable. Each sampling occasion was identified by a date, a year and a district geocode. The best multivariable model was selected using a stepwise backward approach based on the Akaike Information Criterion (AIC). The Area Under the Curve (AUC) of the Receiver Operating Characteristic (ROC) plot was used to estimate the model performance. We also used cross-validation to measure the performance of the best model. Data was randomly split into training (2/3 of data) and test (1/3 of data) sets. Training data is used to produce the prediction model, while the test data is used to test the model performance. Given the size of our dataset, we chose to keep 2/3 of the data in the training set to optimize model performance. We performed repeated cross-validations 1000 times to estimate the mean and standard deviation of the cross-validated AUC (cvAUC) of the best model.

The best model was used to predict leptospirosis infection risk in 2012 and 2016 for three periods (mid-January, mid-May and mid-September) which represents the middle of the dry season, the beginning of the rainy season and the end of the rainy season, respectively for central and northern Thailand.

## Results

**Fig. 1:**
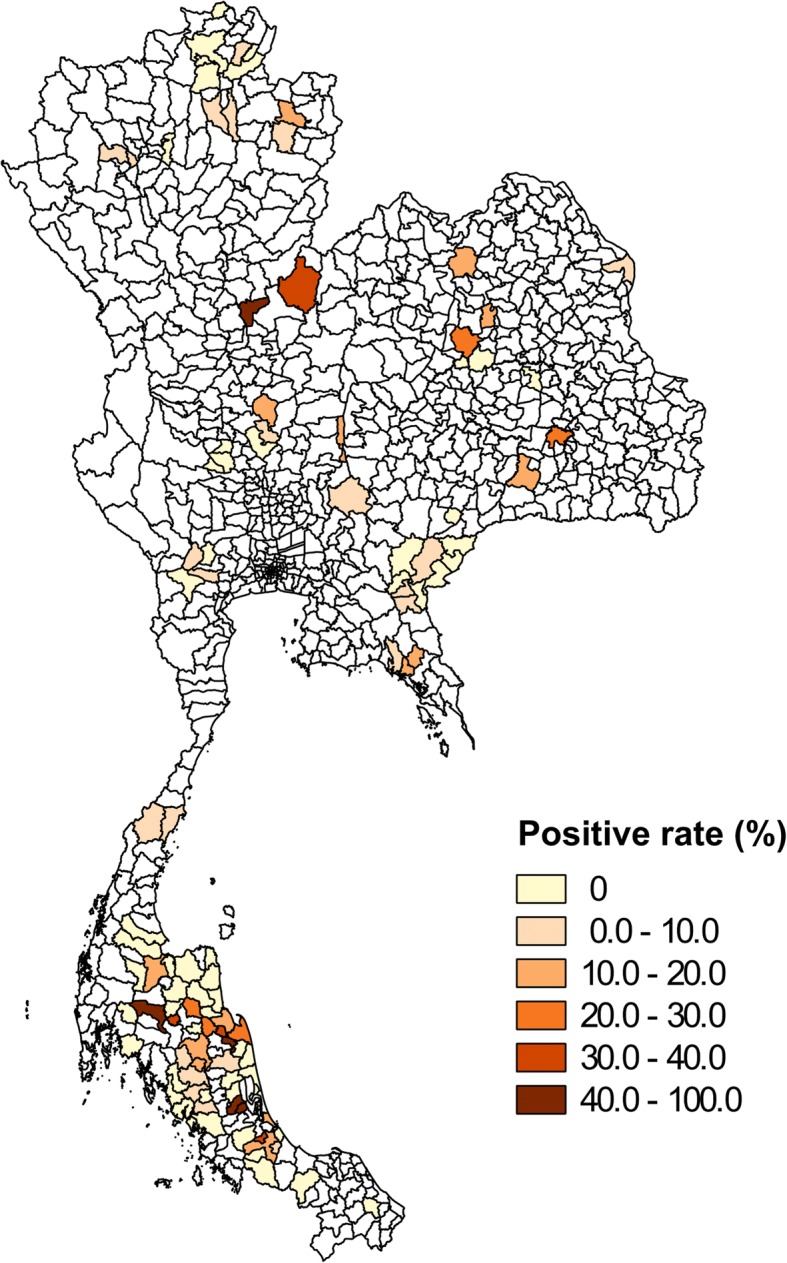
Map of the positive rate of leptospirosis in cattle and buffalo in 107 districts of Thailand. Urine samples were tested by LAMP. The non-sampled districts are presented in white

**Fig. 2:**
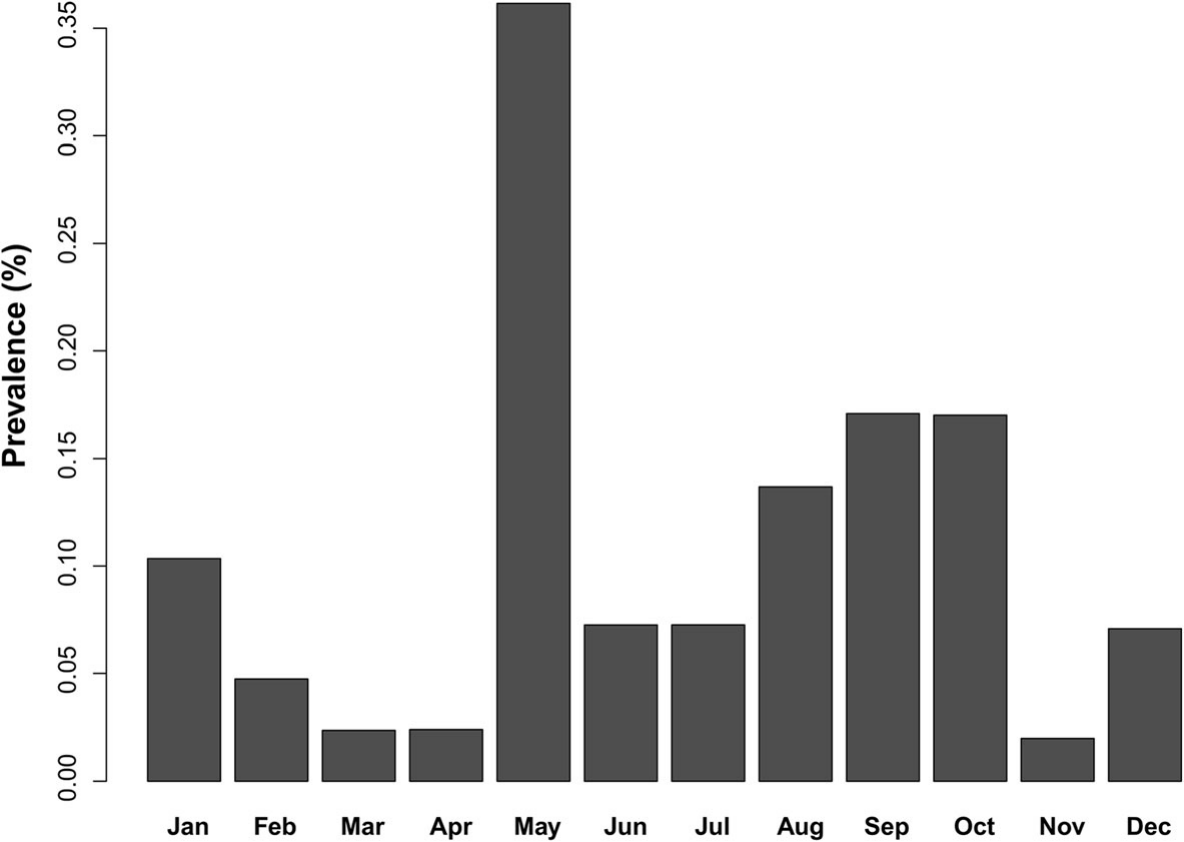
The leptospirosis prevalence observed per month in both cattle and buffalo for 2011–2013

A total of 3571 urine samples of cattle and buffalo were tested by the LAMP technique. 311 samples were positive. The overall uroprevalence over 107 districts is presented in Fig. 1. Positive samples were recorded in 51 districts (47.66% of districts). From the temporal aspect, higher prevalence was observed in May (Fig. 2), which is the beginning of the rainy season in the central and northern part of Thailand [31].

The results of the univariate linear regressions show that the percentage of flooded area and the percentage of flooded area with a 1 month lag were found to be significant (Additional file 1:Table S1). The risk of livestock infection was higher if the percentage of flood area was higher.

**Fig. 3:**
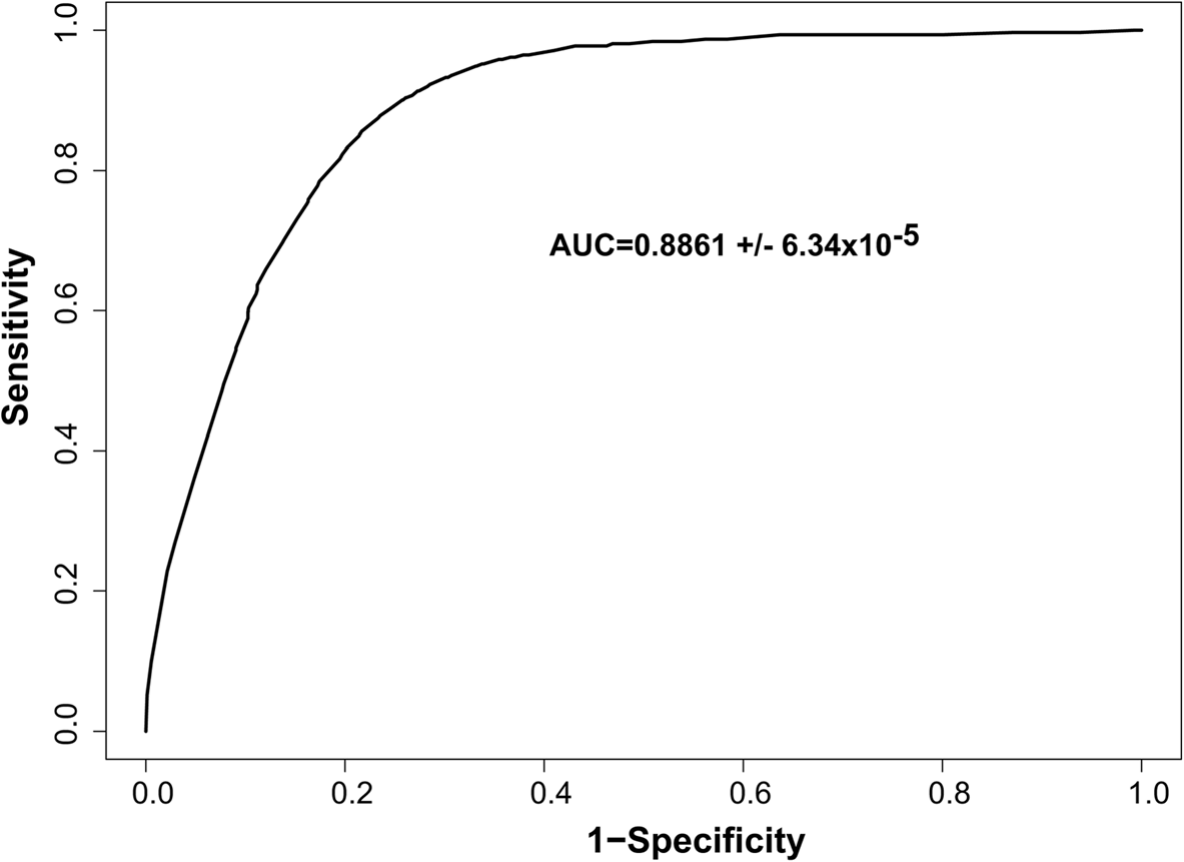
ROC curve of the best generalized linear mixed model

**Fig. 4:**
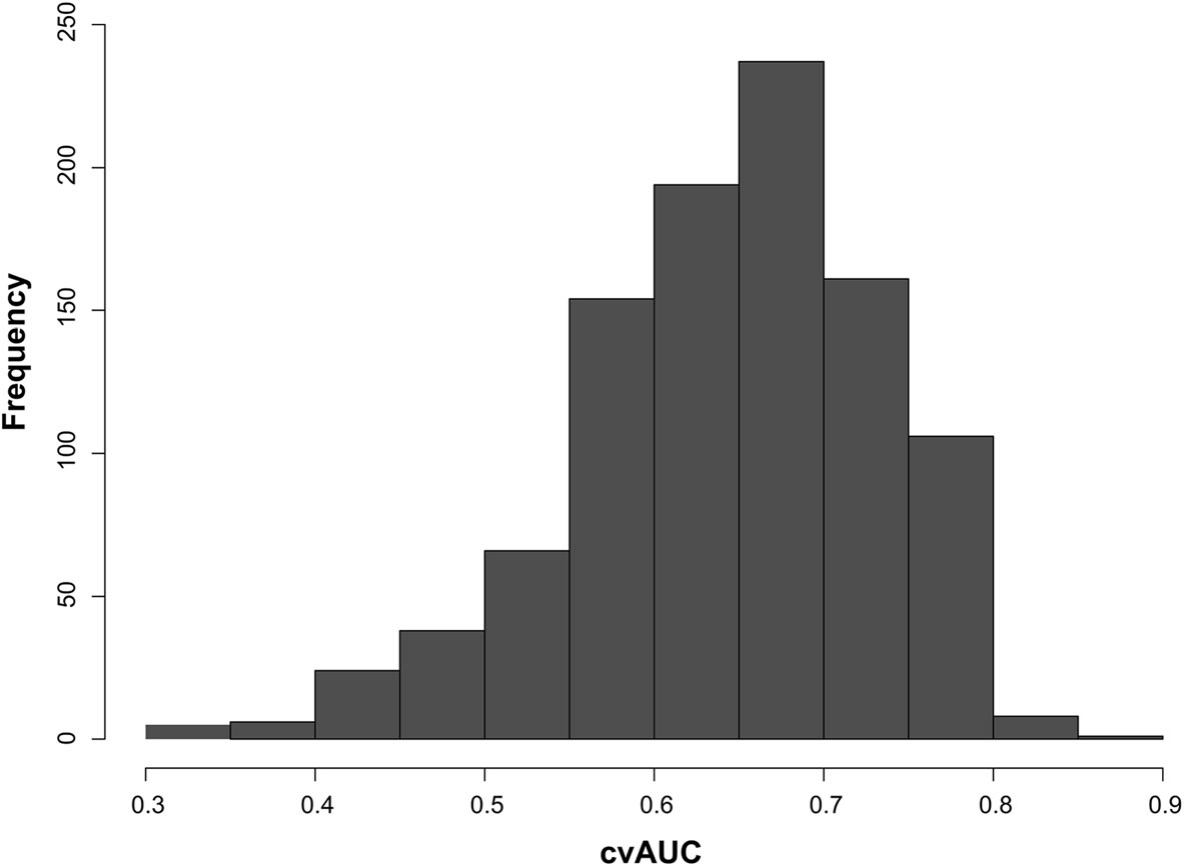
The cross-validated AUC distribution

Three explanatory variables were kept in the final model based on the stepwise backward approach: the percentage of flooded area, human and livestock population densities (Table 1). This final model was applied to predict the risk of *Leptospira* presence at the district level, it showed high performance with an AUC of 0.8861 (Fig. 3). The percentage of flooded area was the only variable significantly associated with the prevalence of leptospirosis in cattle and buffalo in the GLMM (*p* = 0.023, Table 1). The cvAUC had a mean of 0.6427 (sd = 0.0827). The distribution of the 1000 estimations of the cvAUC is shown in Fig. 4.

**Table 1:** Results of the best generalized linear mixed model as selected by a stepwise backward approach with the AIC

Variable	Odd Ratio	95% Confidence Interval	*p*-value
Intercept	0.0309	0.0183–0.0473	<2e-16***
Percentage of flood area	1.5794	1.0611–2.3629	0.023*
Human population density	1.3495	0.9511–1.9016	0.084
Livestock population density	0.5989	0.3079–1.0957	0.105

*p < 0.05, ***p < 0.001

**Fig. 5:**
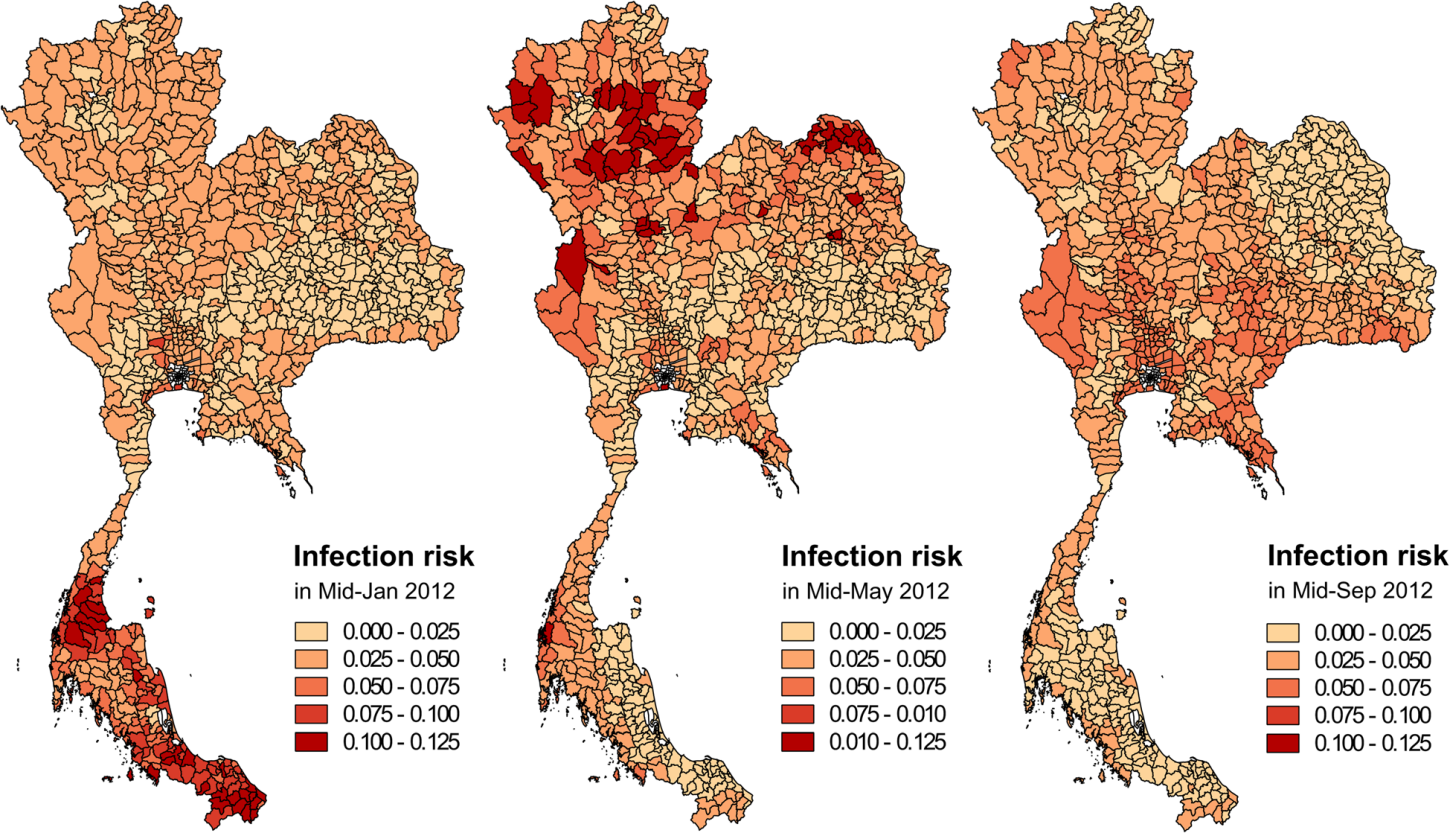
Map of the prediction of leptospirosis infection risk using the final multivariate linear regression model in three different periods of 2012. A leptospirosis infection risk of 0.1 indicates that approximately 1/10 livestock are expected to be positive by LAMP for leptospirosis infection. The non-predicted districts are presented in white

Maps of leptospirosis infection risk were produced from the final model in the middle of January, May, and September in 2012, which corresponds to the period when most data were collected (Fig. 5). As expected from the results of the model, the areas of increased leptospirosis risk vary seasonally (Fig. 5) and are found in the regions with a high percentage of area flooded (Additional file 2: Figure S1). The districts with a high leptospirosis infection risk in mid-January were mostly located in the southern part of Thailand, especially in the south-east coastal regions, i.e. during the high rainfall period in this area (Additional file 2: Figure S2) [31]. In mid-May, high leptospirosis infection risk mostly occurs in northern and northeastern parts, which correspond to the beginning of the rainy season in this part of Thailand. In mid-September, high leptospirosis infection risk areas occurred in all parts except for the southern part, and was particularly high in the central part. In this analysis, the final model was also used to predict the leptospirosis infection risk in 2016 (Additional file 2: Figure S3). The leptospirosis infection risk districts were also mostly found in regions with a high percentage of flooded area (Additional file 2: Figure S4).

## Discussion

This study investigates the relation between cattle and buffalo leptospirosis infections and flooding based on cross-sectional surveillance during 2011–2013 in Thailand. This analysis provides, to our knowledge, the first predictive risk mapping for cattle and buffalo leptospirosis in Thailand. The temporal and spatial variations of leptospirosis infection in Thailand appears to be associated with flooding.

Results of the GLMM show a significant association between the percentage of flood area and leptospirosis infection in cattle and buffalo at the district level. The flooding area was evaluated using a remote sensing indicator [18, 23]. This finding suggests that exposure to flooding increases the risk of leptospirosis infection for cattle and buffalo. Most of the samples used in this study were collected in rural areas. In these areas, the soil may become contaminated with leptospires because of the presence of infected animals. When flooding or heavy rainfall occurs, the water picks up contaminated soil and animal excreta from the soil. This results in the spread of leptospirosis through contaminated water [32, 33]. Flooding could possibly be the principal reason for leptospirosis epidemics above other factors [34]. This is consistent with other studies showing that local flooding can play an important role in leptospirosis transmission [17, 18, 34]. Therefore, flood control could be an option to reduce the risk of leptospirosis infection in animals, which can be a major reservoir for human infection [4, 9].

Furthermore, the results of the univariate linear regressions show that the flooding factor is the only significant factor and is a better indicator than the amount of rainfall and the accumulation of rainfall. It may be because rainfall does not directly influence leptospirosis transmission while flooding facilitating it. Rainfall has previously been associated with leptospirosis but often with a time lag of 1–3 months [35, 36] which is likely the lag between rainfall and flooding. A remotely sensed flooding indicator is likely to be a more accurate predictor of the risk of leptospirosis infection than using rainfall.

The predicted risk maps of leptospirosis infection were created based on the final model for 3 periods in 2012. In each part of Thailand, higher infection risk was observed during the first floods after a dry period in that part of the country. This influence of the first flood of the year has been suggested in other studies [18]. It could be responsible for the rapid dissemination of leptospires concentrated in small areas during the dry season. High prevalence in livestock is not predicted in the same period for the whole Thailand. Three main periods of risk can be identified and associated with three different parts of Thailand (i.e., Northern, Central and Southern parts) and are related with the periods of flooding. The difference in these flooding periods is mainly due to two factors: a) the difference of rainfall seasonality between southern Thailand and the rest of the country, and b) the delay between rainfall and flooding between the central part and the northeastern part of the country. The central part of the country is downstream of the most important rivers in Thailand, and major flooding occurs later than in the rest of the country, in September to November, with an increased intensity. This explains why high risk occurs for most districts in this period, which also corresponds to its high population [12].

With the backward step approach, the final model includes human and livestock population densities. However, the model results show that those variables are not significant. Furthermore, these variables should be interpreted very cautiously because several confounding factors could be involved. Thus, they were kept because they improved the final model (based on the decrease of the AIC), but they should not be over-interpreted.

Our study was based on a cross-sectional survey [4], which was limited as there may be procedural concerns. It does not provide data for all districts in the country and for all seasons in each district. A longitudinal survey is strongly suggested in further studies, with repeated sampling in a larger number of districts in the whole country. It would provide better data to understand the seasonality of leptospirosis infection and could provide a more accurate disease transmission model. The samples in each district were mostly collected only once. However, the samples were distributed over every part of Thailand for all seasons. Furthermore, the model had a relatively good performance (AUC =0.8861) but a lower and quite variable cross-validated AUC (mean cvAUC = 0.6427, sd = 0.0827, Fig. 4). This difference between AUC and cvAUC, and the variability of the cvAUC may be explained by the relatively small size of our dataset at the district level leading to a small validation dataset (71 districts for the training dataset and only 36 for the validation dataset). Furthermore, given this size limit, some validation datasets may include a different proportion of southern districts than their matching training datasets. The difference of flooding patterns between southern Thailand and the rest of the country may then further explain the lower cvAUC. Training the model on a larger dataset and having an independent large dataset to validate it would help build a more robust model.

The presence of pathogenic leptospires in livestock was tested with LAMP [4, 10], which allows a simple and rapid diagnosis of leptospirosis with high accuracy. However, this technique cannot provide any genotypic information, thus could not be used to compare pathogenic strains in the study. However, in Thailand, the accuracy of LAMP (97.0%) was higher than real-time PCR (91.9%) [10]. Thus, results from this technique can be used with confidence in our study to investigate the association of livestock leptospirosis infection with environmental factors.

Other environmental risk factors such as soil type and land use, which were not explored in this study, may be required to better characterize leptospirosis infection risk. A previous study showed that agricultural land and clay loams soil are significantly associated with leptospirosis infection in humans [37]. These factors could influence the identification of high-risk areas and help improve our model.

Other individual variables such as sex and age of the animals investigated were not considered in this study due to data limits. These factors could help us to improve the model and may impact the results [38, 39]. *Leptospira* can infect a wide range of livestock including pigs, goats and sheep [40, 41]. Studies of these animals should also be implemented as they may also contribute to leptospirosis epidemics. However, the present study focused on the flooding indicator associated with cattle and buffalo infection. The good performance of the model shows that flooding is a major factor that should be considered in leptospirosis risk models.

## Conclusion

Our findings could identify flooding as a major driver of the risk of leptospirosis infection in cattle and buffalo. Public awareness about the risk of leptospirosis during flooding should be raised in order for people to take prevention measures when possible. The risk maps could also help to develop effective intervention strategies and optimize the allocation of public health resources, veterinary care and control measures. A high level of livestock infection could increase the risk to human health due to contact with infected animals or a contaminated environment by the urine of infected animals [2, 34]. Livestock may then play an important role as a potential indicator of high-risk areas for leptospirosis in humans. Further study needs to be done to assess the risks associated with contact between livestock and humans. In this regard, further data needs to be collected and made available.

## Additional files

Additional file 1: **Table S1.** Summary results of the univariable linear regression model (with binomial function and random effect). (DOCX 14 kb)

Additional file 2: **Figure S1.** Percentage of flood area in 2012. **Figure S2.** The monthly rainfall of Thailand in 2012. **Figure S3.** Prediction of leptospirosis infection risk in 2016. The non-predicted districts are presented in white. **Figure S4.** Percentage of Flood area in 2016.**Figure S5.** Maps of human density (people/km^2^) and livestock density (animal/km^2^). (DOCX 3416 kb)

## Abbreviations

AIC: Akaike Information Criterion
AUC: Area Under the Curve
GLMM: Generalized linear mixed model
LAMP: Loop-mediated isothermal amplification
MNDWI: Modified normalized difference water index
MODIS: Moderate Resolution Imaging Spectroradiometer
ROC: Receiver Operating Characteristic
SRTM: Shuttle Radar Topographic Mission
TRMM: Tropical Rainfall Measuring Mission

## References

[1] Adler B, de la Peña Moctezuma A (2010). Leptospira and leptospirosis. Vet Microbiol.

[2] Haake DA, Levett PN (2015). Leptospirosis in humans. Leptospira and Leptospirosis edn: Springer.

[3] Cerqueira GM, Picardeau M (2009). A century of Leptospira strain typing. Infect Genet Evol.

[4] Suwancharoen D, Limlertvatee S, Chetiyawan P, Tongpan P, Sangkaew N, Sawaddee Y, Inthakan K, Wiratsudakul A (2016). A nationwide survey of pathogenic leptospires in urine of cattle and buffaloes by loop-mediated isothermal amplification (LAMP) method in Thailand, 2011–2013. J Vet Med Sci.

[5] Salgado M, Otto B, Sandoval E, Reinhardt G, Boqvist S (2014). A cross sectional observational study to estimate herd level risk factors for Leptospira spp. serovars in small holder dairy cattle farms in southern Chile. BMC Vet Res.

[6] Natarajaseenivasan K, Vedhagiri K, Sivabalan V, Prabagaran SG, Sukumar S, Artiushin SC, Timoney JF (2011). Seroprevalence of Leptospira borgpetersenii serovar javanica infection among dairy cattle, rats and humans in the Cauvery river valley of southern India. Southeast Asian journal of tropical Medicineand. Public Health.

[7] Ellis WA, Adler B (2015). Animal Leptospirosis. Leptospira and Leptospirosis. edn.

[8] Department of Livestock Development (DLD), Ministry of Agriculture and Cooperatives of Thailand. Number of farmer households and livestock classified by species in Thailand [in Thai]. 2012. http://ict.dld.go.th. Accessed 19 June 2017.

[9] Chadsuthi S, Bicout DJ, Wiratsudakul A, Suwancharoen D, Petkanchanapong W, Modchang C, Triampo W, Ratanakorn P, Chalvet-Monfray K (2017). Investigation on predominant Leptospira serovars and its distribution in humans and livestock in Thailand, 2010-2015. PLoS Negl Trop Dis.

[10] Suwancharoen D, Sittiwicheanwong B, Wiratsudakul A (2016). Evaluation of loop-mediated isothermal amplification method (LAMP) for pathogenic Leptospira spp. detection with leptospires isolation and real-time PCR. J Vet Med Sci.

[11] Saito M, Villanueva SY, Chakraborty A, Miyahara S, Segawa T, Asoh T, Ozuru R, Gloriani NG, Yanagihara Y, Yoshida S-i (2013). Comparative analysis of Leptospira strains isolated from environmental soil and water in the Philippines and Japan. Appl Environ Microbiol.

[12] Bureau of Epidemiology, DDC, MPH. Leptospirosis. 2017. http://www.boe.moph.go.th/boedb/surdata/disease.php?ds=43. Accessed November 2017.

[13] Kawaguchi L, Sengkeopraseuth B, Tsuyuoka R, Koizumi N, Akashi H, Vongphrachanh P, Watanabe H, Aoyama A (2008). Seroprevalence of leptospirosis and risk factor analysis in flood-prone rural areas in Lao PDR. Am J Trop Med Hyg.

[14] Dechet AM, Parsons M, Rambaran M, Mohamed-Rambaran P, Florendo-Cumbermack A, Persaud S, Baboolal S, Ari MD, Shadomy SV, Zaki SR (2012). Leptospirosis outbreak following severe flooding: a rapid assessment and mass prophylaxis campaign; Guyana, January–February 2005. PLoS One.

[15] Agampodi SB, Dahanayaka NJ, Bandaranayaka AK, Perera M, Priyankara S, Weerawansa P, Matthias MA, Vinetz JM (2014). Regional differences of leptospirosis in Sri Lanka: observations from a flood-associated outbreak in 2011. PLoS Negl Trop Dis.

[16] Della Rossa P, Tantrakarnapa K, Sutdan D, Kasetsinsombat K, Cosson J-F, Supputamongkol Y, Chaisiri K, Tran A, Supputamongkol S, Binot A. Environmental factors and public health policy associated with human and rodent infection by leptospirosis: a land cover-based study in Nan province, Thailand. Epidemiol Infect. 2015:1–13. 10.1017/S0950268815002903 PMC9150581 26607833

[17] Lau CL, Smythe LD, Craig SB, Weinstein P (2010). Climate change, flooding, urbanisation and leptospirosis: fuelling the fire?. Trans R Soc Trop Med Hyg.

[18] Ledien J, Sorn S, Hem S, Huy R, Buchy P, Tarantola A, Cappelle J (2017). Assessing the performance of remotely-sensed flooding indicators and their potential contribution to early warning for leptospirosis in Cambodia. PLoS One.

[19] Yamane T (1973). Statistics: an introductory analysis.

[20] Goddard Earth Sciences Data and Information Services Center. TRMM (TMPA-RT) Near Real-Time Precipitation L3 1 day 0.25 degree × 0.25 degree V7, Greenbelt, MD, Goddard Earth Sciences Data and Information Services Center (GES DISC). 2016. https://disc.gsfc.nasa.gov/datacollection/TRMM_3B42RT_Daily_7.html. Accessed November 2016.

[21] Huffman GJ, Bolvin DT. Real-time TRMM multi-satellite precipitation analysis data set documentation. NASA Tech Doc. 2015.

[22] Huffman GJ, Bolvin DT, Nelkin EJ, Wolff DB, Adler RF, Gu G, Hong Y, Bowman KP, Stocker EF (2007). The TRMM multisatellite precipitation analysis (TMPA): quasi-global, multiyear, combined-sensor precipitation estimates at fine scales. J Hydrometeorol.

[23] Xu H (2006). Modification of normalised difference water index (NDWI) to enhance open water features in remotely sensed imagery. Int J Remote Sens.

[24] Feyisa GL, Meilby H, Fensholt R, Proud SR (2014). Automated water extraction index: a new technique for surface water mapping using Landsat imagery. Remote Sens Environ.

[25] Gautam VK, Gaurav PK, Murugan P, Annadurai M (2015). Assessment of surface water Dynamicsin Bangalore using WRI, NDWI, MNDWI, supervised classification and KT transformation. Aquat Procedia.

[26] Quantum GIS Development Team. Quantum GIS geographic information system v2.8.3. Open source geospatial foundation project. 2015. https://www.qgis.org. Accessed Nov 2016.

[27] Kwak Y, Park J, Fukami K (2014). Estimating floodwater from MODIS time series and SRTM DEM data. Artificial Life and Robotics.

[28] Suwancharoen D, Chaisakdanugull Y, Thanapongtharm W, Yoshida S (2013). Serological survey of leptospirosis in livestock in Thailand. Epidemiology & Infection.

[29] R Core Team. R: a language and environment for statistical computing. Vienna: R Foundation for Statistical Computing; 2014. https://www.r-project.org/. Accessed Nov 2016.

[30] Bates D, Mächler M, Bolker B, Walker S (2015). Fitting linear mixed-effects models using lme4. J Stat Softw.

[31] Thai Meteorological Department. Thailand Annual Weather Summary, 2012. 2012. https://www.tmd.go.th/programs/uploads/yearlySummary/Annua_2012-1.pdf. Accessed 19 June 2017.

[32] Socolovschi C, Angelakis E, Renvoisé A, Fournier P-E, Marié JL, Davoust B, Stein A, Raoult D (2011). Strikes, flooding, rats, and leptospirosis in Marseille, France. Int J Infect Dis.

[33] Brockmann S, Piechotowski I, Bock-Hensley O, Winter C, Oehme R, Zimmermann S, Hartelt K, Luge E, Nöckler K, Schneider T (2010). Outbreak of leptospirosis among triathlon participants in Germany. 2006 BMC Infectious Diseases.

[34] Mwachui MA, Crump L, Hartskeerl R, Zinsstag J, Hattendorf J (2015). Environmental and behavioural determinants of leptospirosis transmission: a systematic review. PLoS Negl Trop Dis.

[35] Desvars A, Jégo S, Chiroleu F, Bourhy P, Cardinale E, Michault A (2011). Seasonality of human leptospirosis in Reunion Island (Indian Ocean) and its association with meteorological data. PLoS One.

[36] Robertson C, Nelson TA, Stephen C (2011). Spatial epidemiology of suspected clinical leptospirosis in Sri Lanka. Epidemiol Infect.

[37] Lau CL, Clements AC, Skelly C, Dobson AJ, Smythe LD, Weinstein P (2012). Leptospirosis in American Samoa–estimating and mapping risk using environmental data. PLoS Negl Trop Dis.

[38] Alton GD, Berke O, Reid-Smith R, Ojkic D, Prescott JF (2009). Increase in seroprevalence of canine leptospirosis and its risk factors, Ontario 1998–2006. Can J Vet Res.

[39] Ayral F, Artois J, Zilber AL, WidÉN F, Pounder KC, Aubert D, Bicout DJ, Artois M (2014). The relationship between socioeconomic indices and potentially zoonotic pathogens carried by wild Norway rats: a survey in Rhône, France (2010–2012). Epidemiol Infect.

[40] Martins G, Lilenbaum W (2013). The panorama of animal leptospirosis in Rio de Janeiro, Brazil, regarding the seroepidemiology of the infection in tropical regions. BMC Vet Res.

[41] Suepaul SM, Carrington CV, Campbell M, Borde G, Adesiyun AA (2011). Seroepidemiology of leptospirosis in livestock in Trinidad. Trop Anim Health Prod.

