# Supplementary material for: A remotely sensed flooding indicator associated with cattle and buffalo leptospirosis cases in Thailand 2011–2013
Source: BMC Infect Dis. 2018 Nov 29;18:602. doi: 10.1186/s12879-018-3537-3 (PMC6267035; doi:10.1186/s12879-018-3537-3)
Supplement: Supplementary file 1 — Table S1. Summary results of the univariable linear regression model (with binomial function and random effect). (DOCX 14 kb) [file 12879_2018_3537_MOESM1_ESM.docx]

**Additional file 1:**

**Table S1.** **Summary results of the univariable linear regression model (with binomial function and random effect).**

| **Variable** | **Odd Ratio** | **95% Confidence Interval** | **p-value** |
| --- | --- | --- | --- |
| Amount of rainfall at sampling day | 0.9378 | 0.6588 - 1.2861 | 0.707 |
| Cumulative of rainfall for 30 days | 1.1205 | 0.8121 - 1.5020 | 0.466 |
| Percentage of flood area | 1.7129 | 1.1383 - 2.5942 | 0.009** |
| Percentage of flood area at 1 month lag | 1.4770 | 1.0034 - 2.1994 | 0.047* |
| Average elevation | 1.0118 | 0.6645 - 1.5262 | 0.966 |
| Human population density | 1.3258 | 0.9134 - 1.9130 | 0.133 |
| livestock population density | 0.6022 | 0.3082 - 1.1141 | 0.113 |

*p<0.05, **p<0.01
