# Supplementary material for: A remotely sensed flooding indicator associated with cattle and buffalo leptospirosis cases in Thailand 2011–2013
Source: BMC Infect Dis. 2018 Nov 29;18:602. doi: 10.1186/s12879-018-3537-3 (PMC6267035; doi:10.1186/s12879-018-3537-3)
Supplement: Supplementary file 2 — Figure S1. Percentage of flood area in 2012. Figure S2. The monthly rainfall of Thailand in 2012. Figure S3. Prediction of leptospirosis infection risk in 2016. The non-predicted districts are presented in white. Figure S4. Percentage of Flood area in 2016.Figure S5. Maps of human density (people/km2) and livestock density (animal/km2). (DOCX 3416 kb) [file 12879_2018_3537_MOESM2_ESM.docx]

**Additional file 2:**

**
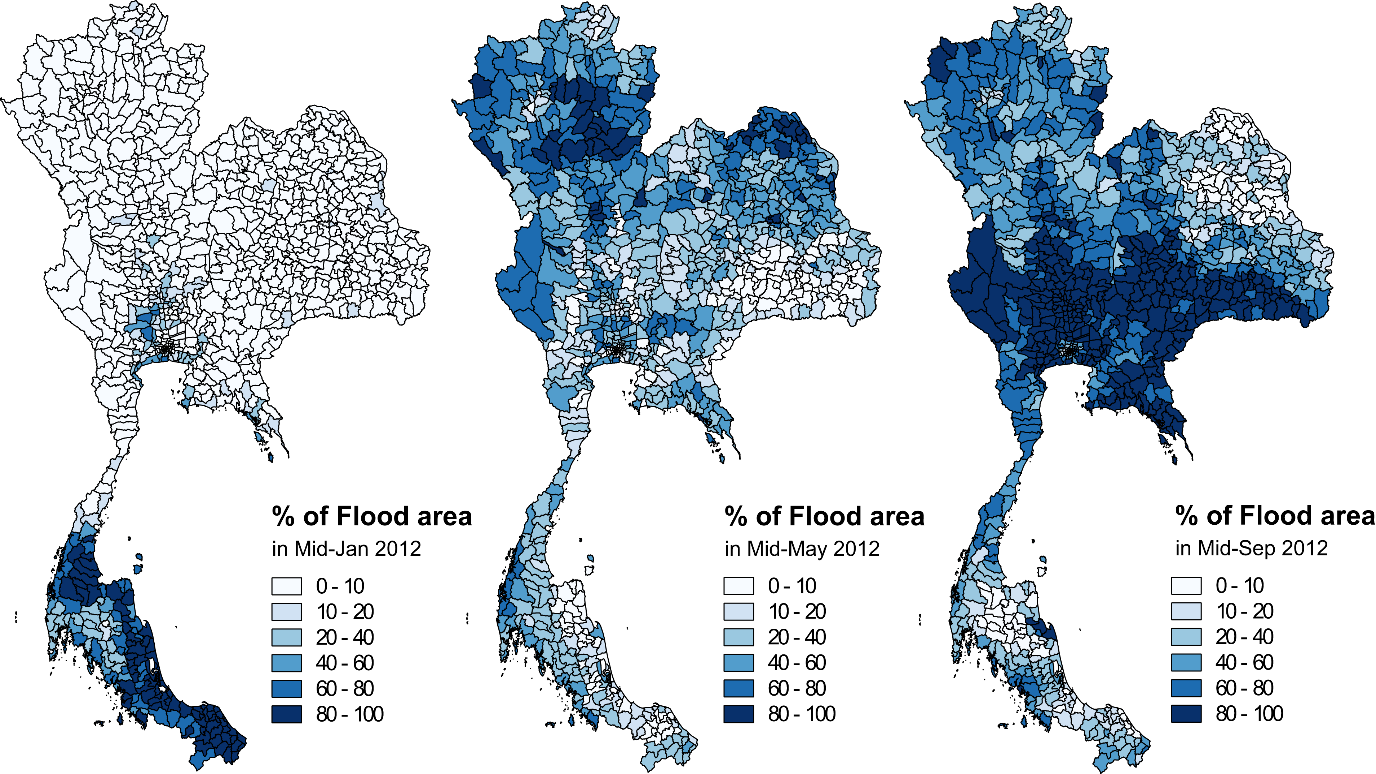
**

**Figure S1. Percentage of flood area in 2012.**

**
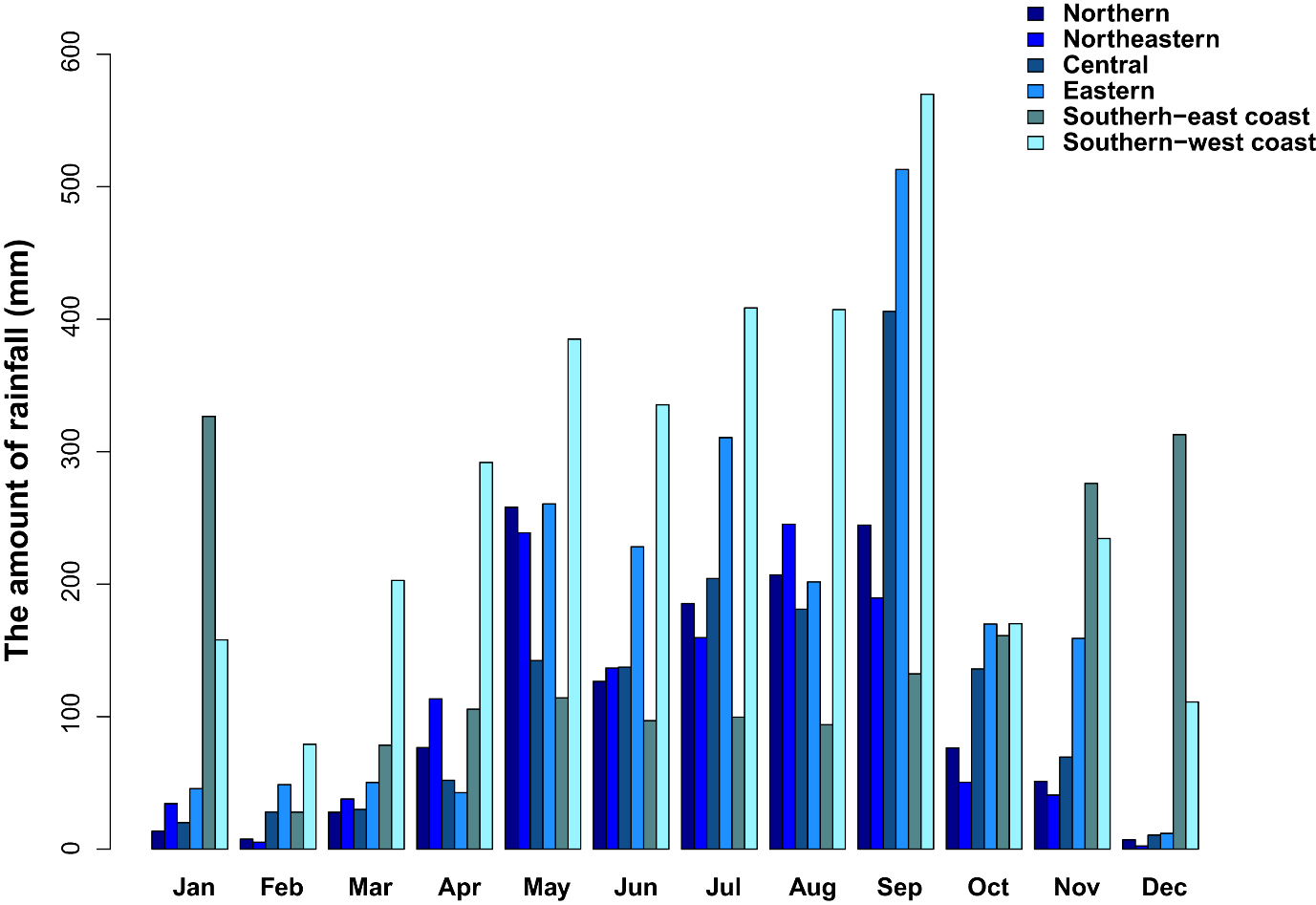
**

**Figure S2. The monthly rainfall of Thailand in 2012.**

**
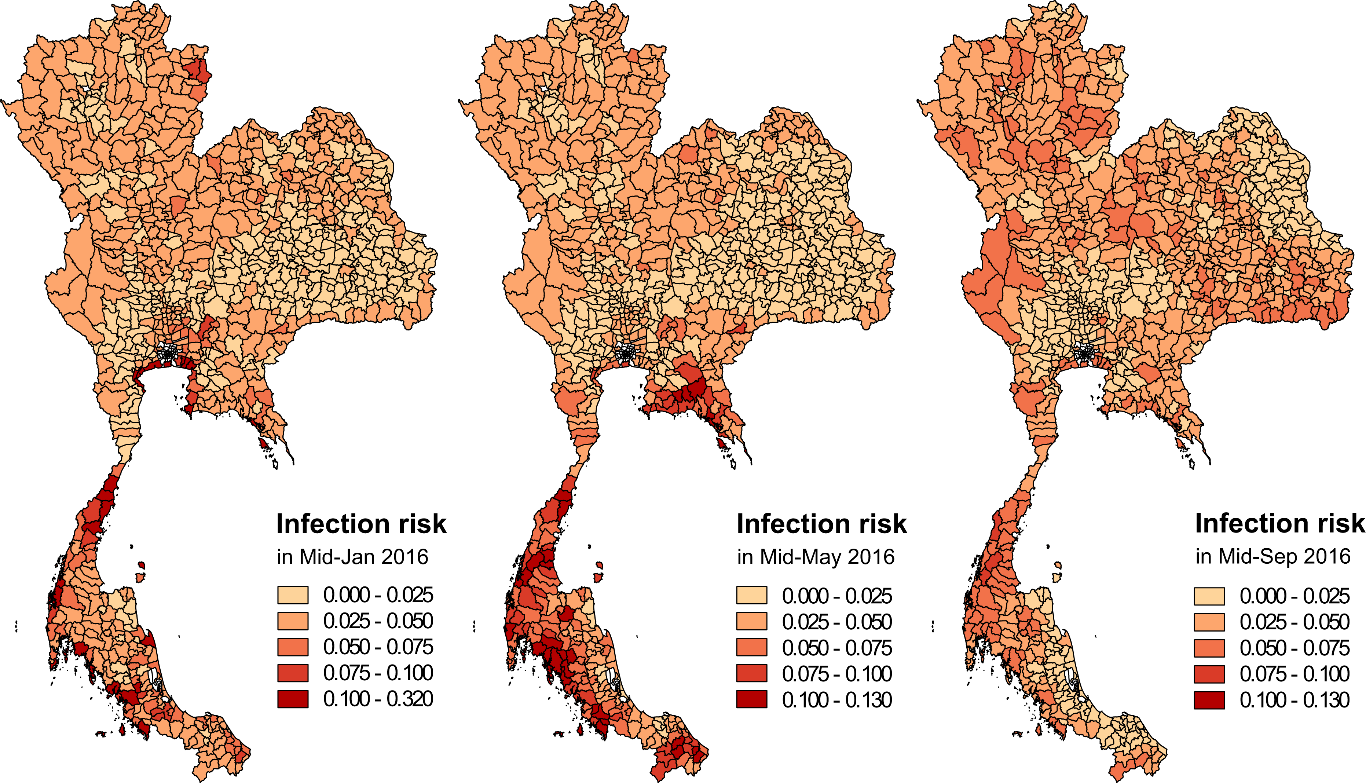
**

**Figure S3. Prediction of leptospirosis infection risk in 2016. The non-predicted districts are presented in white.**

**
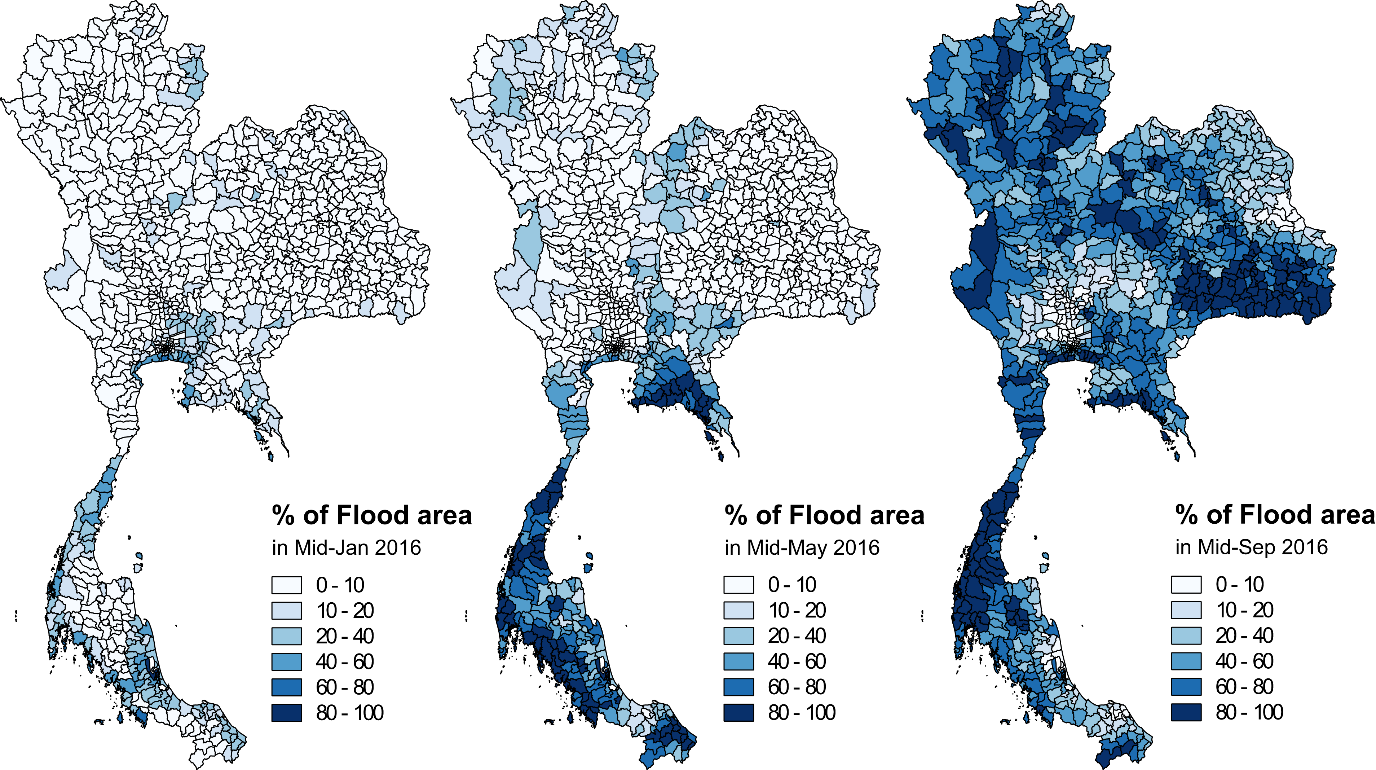
**

**Figure S4. Percentage of Flood area in 2016.**

**
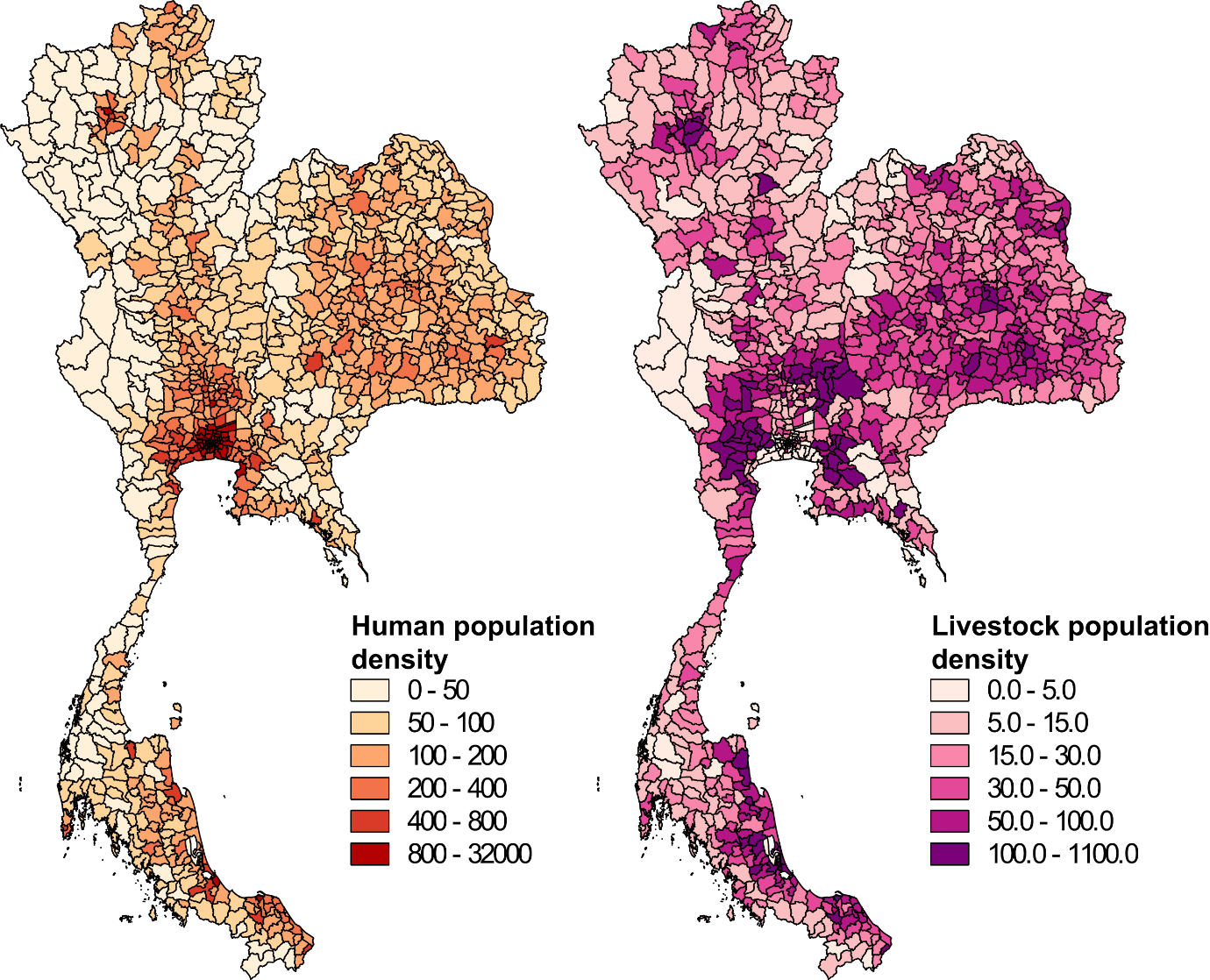
**

**Figure S5. Maps of human density (people/km^2^) and livestock density (animal/km^2^).**
